# Systematic Review and Meta-Analysis of *Campylobacter* Species Contamination in Poultry, Meat, and Processing Environments in South Korea

**DOI:** 10.3390/microorganisms11112722

**Published:** 2023-11-07

**Authors:** Hyeon Ji Je, Saloni Singh, Dong Woo Kim, Hyun Seok Hur, Ah Leum Kim, Eun Jin Seo, Ok Kyung Koo

**Affiliations:** 1 Department of Food Science & Technology, Chungnam National University, Daejeon 34134, Republic of Koreakdw8125@hanmail.net (D.W.K.); hstone@krict.re.kr (H.S.H.); 202250416@o.cnu.ac.kr (A.L.K.); 2 Agro-Bioproduct Analysis Team, Korea Agriculture Technology Promotion Agency, Iksan 54667, Republic of Korea; seoej1997@naver.com

**Keywords:** *Campylobacter jejuni*, *Campylobacter coli*, prevalence, cross-contamination, foodborne-pathogens

## Abstract

*Campylobacter* spp. constitute a significant global threat as a leading cause of foodborne illnesses, with poultry meat as a prominent reservoir for these pathogens. South Korea is known for its diverse poultry consumption habits, and continuous outbreaks make it a matter of concern to perform a meta-analysis to identify the primary source of contamination. This systematic review and meta-analysis aimed to assess and compare the prevalence of *Campylobacter* in various poultry and meat types while also considering the importance of environmental factors in South Korea. The meta-analysis revealed that duck meat exhibited the highest prevalence of *Campylobacter*, with a pooled estimate of 70.46% (95% CI: 42.80% to 88.38%), followed by chicken meat at a pooled prevalence of 36.17% (95% CI: 26.44% to 47.91%). Additionally, our analysis highlighted the predominance of *C. jejuni* and *C. coli* in South Korea. These findings underscore the importance of implementing rigorous food safety measures and establishing robust surveillance programs in the poultry industry to mitigate the risk of *Campylobacter*-related foodborne illnesses associated with meat consumption in South Korea.

## 1. Introduction

*Campylobacter* is a Gram-negative, spiral-shaped, and microaerophilic pathogen commonly associated with foodborne illnesses. The optimal growth temperature range for *Campylobacter* spp. is 37–42 °C, which is close to the body temperature of warm-blooded animals [1]. The *Campylobacter* genus comprises 15 known species, and 12 have been linked to causing diseases in humans [2]. Notably, *C. jejuni* and *C. coli* account for over 95% of human *Campylobacter infections* [3]. *Campylobacter* infection can lead to long-term complications such as irritable bowel syndrome (IBS), arthritis, and Guillain–Barré Syndrome (GBS). It is estimated that 0.2 to 1.7 per 1000 individuals with diagnosed or undiagnosed *Campylobacter* infections ultimately develop GBS, accounting for 5–41% of total GBS cases [4].

*C. jejuni* contamination has emerged as a global concern, as evidenced by a comprehensive epidemiological study conducted by Kaakoush et al., 2015 [5]. The study revealed a concerning increase in cases in North America, Europe, and Australia. Furthermore, data from Africa, Asia, and the Middle East indicated a particularly high prevalence among children [6]. In the United States, the Foodborne Disease Active Surveillance Network (FoodNet) reported an annual incidence of approximately 20 cases per 100,000 individuals [7]. An outbreak of *C. jejuni* foodborne infection in 2017 in Seoul, South Korea, was associated with cross-contamination through sharing cutting boards and knives with various food items. Notably, chicken was identified as the primary source, and the bacterium was subsequently transferred to other foods, leading to a widespread outbreak [8]. Another study by Yu et al., 2010, indicated an outbreak in a middle school linked to undercooked chicken as the primary source and subsequently transferred to other foods, leading to a widespread outbreak. [9].

The upswing in foodborne *Campylobacter* infections can be attributed to various intertwined factors. Changes in food production and consumption patterns, including a surge in demand for convenience foods like poultry products, particularly chicken, and a growing tendency to eat out have bolstered *Campylobacter* infections [10]. This bacterium often contaminates chicken products and can spread through cross-contamination in both domestic and commercial kitchens [11]. The emergence of antibiotic-resistant *Campylobacter* strains further complicates treatment and prolongs illness [12]. The global movement of food and people facilitates the spread of *Campylobacter*, leading to sporadic outbreaks and widespread infections [13]. Environmental influences, such as climate change and weather conditions, also affect the prevalence of *Campylobacter* in the environment, adding to the complexity of addressing this public health challenge [14].

Analyzing the historical data allows health authorities and researchers to gain insights into the epidemiology of the disease, such as identifying high-risk areas, vulnerable populations, and seasonal variations [15]. *Campylobacter* outbreaks, despite frequent occurrences, have historically been underreported. However, an observable upward trend in their prevalence has become evident. According to the CDC, from 2004 to 2009, an average of 22 outbreaks were officially reported annually. This figure slightly increased to 31 outbreaks from 2010 to 2012 before declining to 29 from 2013 to 2017 [16]. One of the most significant case studies of Campylobacteriosis was in June 2019, when Askøy in Norway was struck by a significant waterborne outbreak, resulting in over 1500 cases of Campylobacteriosis [17]. Another large-scale outbreak was in New Zealand in 2020, stemming from a contaminated water supply, which led to an estimated 8320 cases [18], underscoring the urgency of addressing this issue globally. According to data published by the Ministry of Food and Drug Safety in South Korea, *Campylobacter* ranks as the third most prevalent food pathogen, following pathogenic *E. coli* and *Salmonella* in this decade [19]. Thus, by examining the patterns and trends of past cases, we can identify common factors, potential sources, and contamination pathways associated with Campylobacteriosis. This analysis offers crucial insights into the causes of contamination and transmission pathways, facilitating evidence-based interventions and strategies to control the disease and protect public health.

Meta-analysis with systematic reviews can offer a comprehensive perspective by amalgamating data from numerous studies and identifying knowledge gaps [20]. Systematic review employs a comprehensive and structured approach to synthesize existing research, while meta-analysis employs statistical methods to combine the outcomes of multiple studies, yielding an overall estimate of the effect of an intervention [21]. These methodologies are crucial for conducting a thorough and exhaustive evaluation of the available research on a specific topic by facilitating the consolidation and synthesis of evidence from diverse studies to enhance the statistical power and generalizability of the findings. By providing a robust summary of the available evidence, they support evidence-based decision-making processes and inform policy formulation and implementation [22]. Ultimately, these approaches benefit researchers, policymakers, clinicians, and other stakeholders by offering a reliable and evidence-based foundation for decision making and further investigation. Therefore, investigating these methodologies would be valuable in guiding future research and informing public health policies and interventions to mitigate the burden of *Campylobacter*-related illness in Korea.

Several studies have been conducted in South Korea to investigate the prevalence of *Campylobacter* contamination in various poultry and meat products. However, these studies have been limited in scope and have reported conflicting results, potentially because of differences in study design, sampling methods, or laboratory testing procedures. Despite efforts to mitigate *Campylobacter* infection in meat products by implementing food safety regulations and guidelines for handling and processing, concerns regarding the prevalence of contamination persist [23]. Therefore, gathering and analyzing all available data from previous studies becomes imperative to facilitate further research in this area. This study aims to determine the prevalence of *Campylobacter* spp. in poultry and meat products in South Korea. This study also aims to consider the environmental conditions under which the products were processed, as these factors may also play a significant role in meat contamination. By conducting a comprehensive analysis of existing studies, this research endeavors to provide a consolidated and robust assessment of the prevalence of *Campylobacter* contamination in poultry and meat products in South Korea, accounting for relevant environmental factors.

## 2. Materials and Methods

### 2.1. Search Strategy

This systematic review strictly adhered to the PRISMA 2020 guidelines (Preferred Reporting Items for Systematic Reviews and Meta-Analysis, http://www.prisma-statement.org/, accessed on 14 March 2023). PRISMA 2020 guidelines were specifically employed for “new systematic reviews which included searches of databases and registers only.” The implementation of PRISMA 2020 aimed to uphold high reporting standards and minimize bias in the review’s findings [24]. Thus, we meticulously followed the PRISMA 2020 guidelines to ensure the transparency, reliability, and rigor of our methodology.

In order to compile a comprehensive body of literature, an exhaustive search was conducted across multiple databases. The search encompassed two widely recognized English databases, Web of Science and PubMed. Additionally, to include relevant studies in South Korea, three Korean-language-based databases were explored: DBpia (https://www.dbpia.co.kr/, accessed on 7 March 2023), RISS (http://www.riss.kr/index.do, accessed on 7 March 2023) and ScienceON (https://scienceon.kisti.re.kr/, accessed on 7 March 2023).

The search algorithm used was “*Campylobacter*” and “Korea”. After retrieving research from each database, the reference management software EndNote 20 (Clarivate Analytics, Boston, MA, USA) was employed to facilitate the de-duplication and screening processes in March 2023.

### 2.2. Eligibility Criteria

A two-level screening procedure was conducted from March to April 2023: title comprisal and abstract screening (Level 1), followed by full-text screening (Level 2). Various criteria aligned with the study’s specific objectives were carefully considered during the data screening and selection process at the searching stage. The authors (HJ Je, DW Kim, HS Hur, AL Kim, and EJ Seo) independently conducted the selection process, rigorously applying the predetermined criteria to each retrieved article. The data were assembled in a Microsoft Excel sheet, and screening was performed according to the parameters set for exclusion and inclusion criteria. In cases where discrepancies in the selection arose, all authors engaged in constructive discussions to reach a consensus, ensuring a meticulous and unbiased assessment of the data.

### 2.3. Inclusion Criteria

The inclusion criteria encompassed studies investigating the presence and contamination of *Campylobacter* in poultry and meat products (chicken, duck, beef, and pork), and contamination by environmental sources (feces, washing water, and equipment). Additionally, articles unrelated to the prevalence, including those centered on antimicrobial research, detection methods, risk analysis, pathogenesis, and other microbiological studies, were excluded. No restrictions were set on the year of publication or the study period; however, articles not in Korean or English were excluded during the initial screening phase. Meticulously and independently, the authors cross-checked each article’s eligibility based on the predefined criteria, ensuring consistency in the selection process. Ultimately, only articles meeting the specific inclusion criteria were considered for this study, and their relevant details were diligently recorded systematically.

### 2.4. Exclusion Criteria

Exclusion criteria in this study were research articles that did not demonstrate the prevalence of *Campylobacter*. Additionally, studies focusing on other bacterial contaminations such as other food products, detection methods different from standard methods, antimicrobial research, and abstract-only papers were excluded. The detection methods excluded from this study were detection via PCR and metagenome analysis without any enrichment process. Furthermore, sampling sites outside South Korea and studies published in languages other than Korean or English were also excluded, but no limitation was made regarding publication years.

### 2.5. Data Extraction

In order to ensure accuracy and reliability, data extraction was carried out by employing a consensus-based approach to minimize the potential for individual bias and enhance the overall quality of the systematic review. Authors (HJ Je, S Singh) extracted data including the sampling period; food type; environmental factors; and the presence of *Campylobacter* spp., *C. jejuni*, or *C. coli* and summarized them in the Microsoft Office Excel software 365, version 2016 (Microsoft Corporation, Redmond, WA, USA). Samples were classified into two groups: food (raw chicken, duck, beef, pork, ham, and meat products) and environmental factors (feces, washing water, and equipment) for meta-analysis.

### 2.6. Risk of Bias for Quality Assessment

A risk of bias assessment was conducted using a questionnaire approach, with scores calculated based on the answers. Each selected study was evaluated based on specific questions, and scores were assigned accordingly (2 points for “YES,” 0 points for “NO,” and 1 point for “UNSURE”) [25]. The total scores ranged from 0 to 12, with scores ≥9 considered high quality, scores ≥6 considered moderate quality, and less than 6 considered low-quality studies [26,27]. The questions were as follows:

Q1. Was the research question/objective clearly described and stated?
Q2. Was the period of study clearly stated?
Q3. Was the sample population clearly specified?
Q4. Was the sampling method described in detail?
Q5. Was the same laboratory method used for all samples in the study?
Q6. Was the isolation method tested based on a standard bacteriological and/or molecular procedure?

### 2.7. Data Analysis

Statistical analysis was performed using the Comprehensive Meta-Analysis Software program version 4 (Biostat Inc., Englewood, NJ, USA). The prevalence of *Campylobacter* and corresponding 95% confidence intervals (CIs) were calculated based on the total number of tested and positive samples. A forest plot was generated to visualize the estimated prevalence and distribution for individual studies and the pooled study estimate within the 95% confidence interval. A random effects model was employed for the meta-analysis, which accounts for expected heterogeneity among the included studies. Heterogeneity levels were assessed using Cochran’s Q statistic and the I-squared (I^2^) inconsistency index. Heterogeneity levels of I^2^ were categorized as low (less than 40%), moderate (between 25% and 50%), substantial (between 50% and 90%), and considerable (greater than 75%) heterogeneity [28].

The groups considered for the study included different types of meat, including beef, pork, chicken, and duck. Since environmental factors play an important role in contamination, various factors like feces, equipment, and washing water were also considered. Equipment includes bedding for cattle, chopping boards, drawers, and knives. The data were also divided into specific detection values for *C. jejuni* and *C. coli* to find which species had more prevalence. The study also included the detection method of using enrichment and selective media techniques.

Publication bias was evaluated using a funnel plot, which could indicate the asymmetrical distribution of effect sizes and standard errors, suggesting the presence of publication bias. Statistical significance for publication bias was determined using a threshold of *p* < 0.05 [29,30].

## 3. Results

### 3.1. Search Results and Risk of Bias

**Figure 1:**
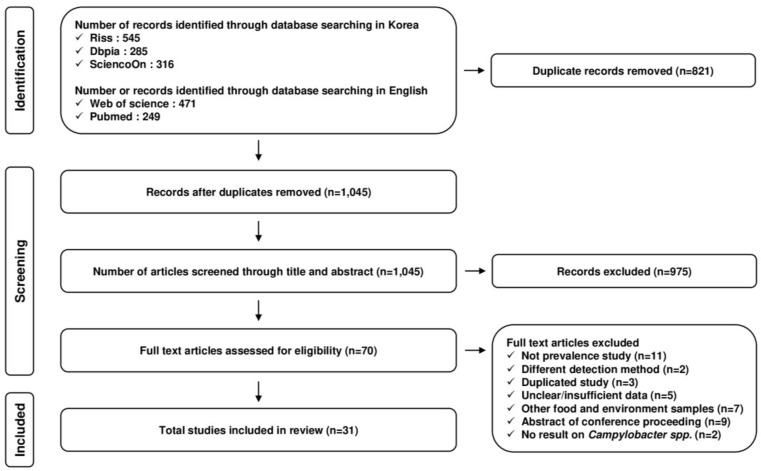
Flowchart of the study selection process followed by a PRISMA 2020 flow diagram for systematic reviews.

In this study, a total of 1045 studies were considered from the databases RISS, DBpia, and Science ON in Korean search engines and Web of Science and PubMed in international search engines after duplicate removal (Figure 1). Title and abstract screening was performed thereafter, resulting in 70 full-text articles. After the full-text screening, 31 studies between 1985 to 2020 were considered for further systematic review and meta-analysis (Table 1). The studies considered in the meta-analysis were confirmed as high (22/32) to moderate (10/32) quality studies, with no low (0/32) quality studies using risk of bias assessment (Figure S1).

### 3.2. Overall Meta-Analysis

**Figure 2:**
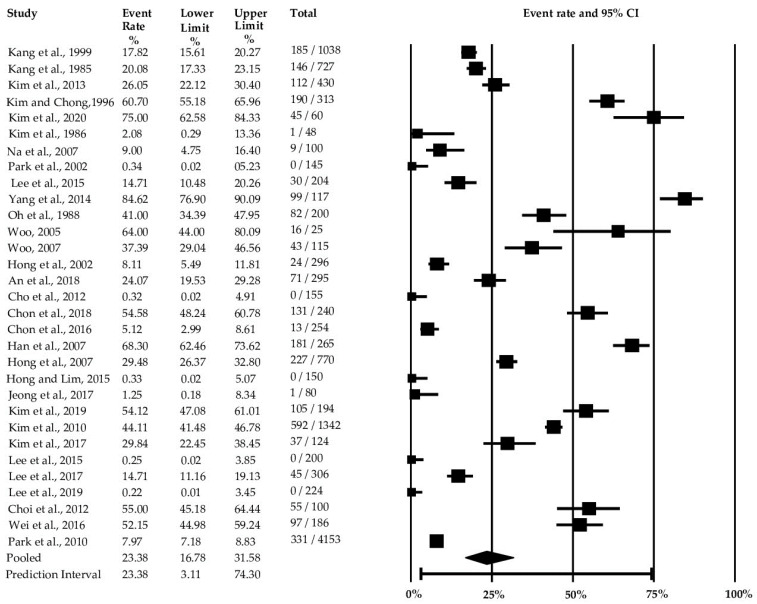
Forest plot of the overall study for the prevalence of *Campylobacter* in South Korea [23,31,32,33,34,35,36,37,38,39,40,41,42,43,44,45,46,47,48,49,50,51,52,53,54,55,56,57,58,59,60].

**Figure 3:**
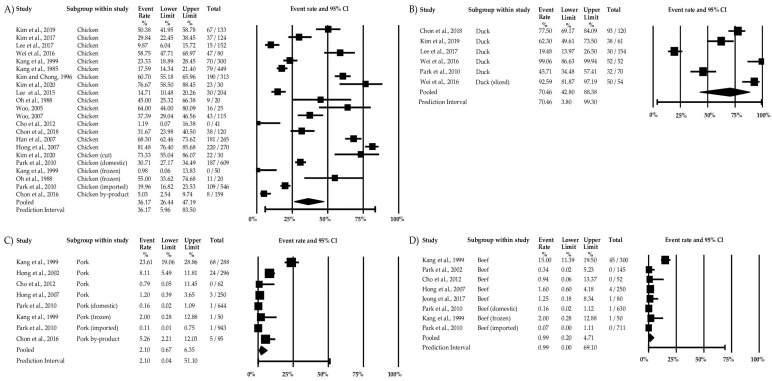
Forest plot of each food type for the prevalence of *Campylobacter* in South Korea: (**A**) chicken, (**B**) duck, (**C**) pork, and (**D**) beef [3,31,32,34,35,39,41,42,43,46,47,48,49,50,52,53,54,57,59,60].

The comprehensive meta-analysis considered all the relevant food and environmental factors. Among the 31 studies, the overall pooled prevalence of *Campylobacter* was 23.38% (95% CI: 16.78–31.58%) (Figure 2 and Figure S2). The analysis showed an I^2^ value of 98% (*p* < 0.001), indicating significant variability among the studies (Table 2). When considering the food groups, ducks exhibited the highest prevalence of *Campylobacter* spp. at 70.46% (95% CI: 42.80–88.38%), followed by chicken with a prevalence rate of 36.17% (95% CI: 26.44–47.19%), pork at 2.10% (95% CI:0.67–6.35%), and beef at 0.99% (95% CI: 0.20–4.71%) (Figure 3 and Figure S3 and Table 2). The analysis also included ham and meat products such as patties, meatballs, and cutlets; however, they did not yield enough studies for meta-analysis.

### 3.3. Campylobacter Prevalence in Food

Studies examining the prevalence of *Campylobacter* species in food sources, particularly poultry products, have consistently found *C. jejuni* to be more prevalent than *C. coli* [61]. Our study verified these findings, as *C. jejuni* exhibited higher prevalence rates than *C. coli* across all samples (Table 3 and Table 4).

### 3.4. Environmental Factors Play a Major Role in Contamination

**Figure 4:**
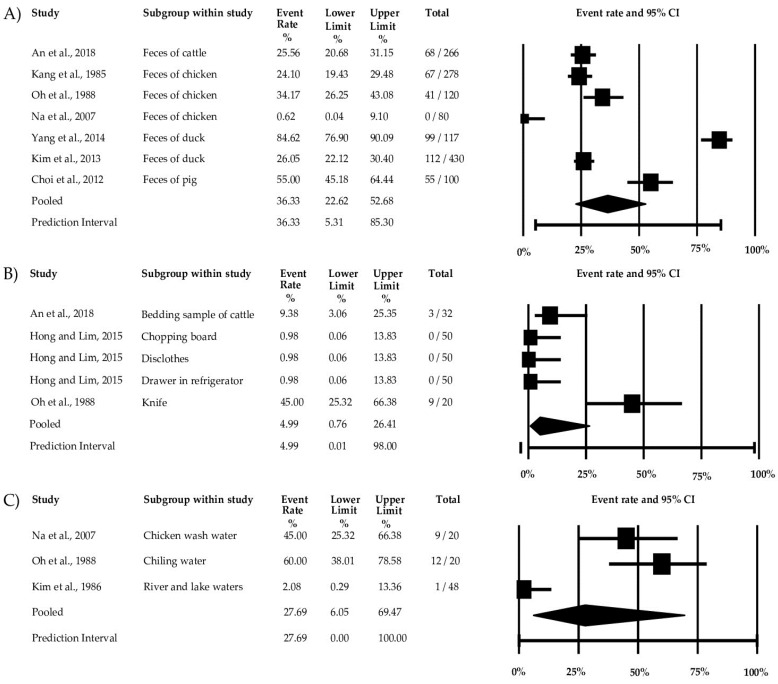
Forest plot of prevalence of *Campylobacter* considering environmental factors and processing environments in South Korea: (**A**) feces, (**B**) equipment, (**C**) wash water [32,33,36,37,40,41,45,51,58].

Among the environmental factors considered in this study, feces showed the highest prevalence at 36.33% (95% CI: 22.62–52.68%), followed by wash water at 27.69% (95% CI: 6.0.5–69.47%) and equipment at 4.99% (95% CI: 0.76–26.41%). Duck feces exhibited the highest prevalence of *Campylobacter* spp., followed by pig and chicken feces. Chilling and chicken wash water also showed high prevalence rates of 60% and 45%, respectively. Among the equipment commonly used, knives showed the highest prevalence at 45% (95% CI: 25.32–63.38%) (Figure 4).

## 4. Discussion

A World Health Organization (WHO) report states that poultry, including chicken and turkey, is a common source of foodborne pathogens such as *Salmonella* and *Campylobacter* [62]. Meat products such as beef and pork are potential sources of *Campylobacter* contamination [63]. Taremi et al. (2006) found the highest prevalence of *Campylobacter* in chicken (63%) and beef (10%) [64]. Given that chicken is the most consumed meat worldwide [65], addressing the prevalence and consequences of *Campylobacter* infections in poultry becomes paramount. Furthermore, *Campylobacter* prevalence is not confined to poultry and meat; it has been found in vegetables, fruits, and fresh produce at an estimated prevalence of approximately 0.53% [66].

During the screening process, the detection method for *Campylobacter* was also considered (Table 1). Although specific differences exist in overall protocols for detecting *Campylobacter*, the methodology was similar in media composition and temperature, which can be excluded from the potential cause of heterogeneity and bias. Components such as amphotericin, sodium bisulfite, sodium pyruvate, and sodium chloride were prevalent across most compositions, with pH levels ranging from 7.2 to 7.4. Additionally, we conducted a risk of bias assessment to determine the quality of the studies considered. Overall, 22 out of 32 studies were classified as high quality, and the remaining nine were moderate quality, without any studies considered low quality (Figure S1).

It is noteworthy that our findings showed a higher prevalence of duck, in contrast to studies conducted in the US (12.5%), UK (50.7%), and Ireland (45.8%) [59]. There could be several factors contributing to a high prevalence of duck, including contamination in duck farms [67], high intestinal concentration, or the protective effects of thicker skin layers [68]. Another explanation is that chicken has recently been the focus of contamination prevention efforts, which may not be the case for ducks [59]. Nevertheless, chicken is still more prevalent than other meat, such as beef and pork. It is important to note the limitations in conducting subgroup analysis due to insufficient study information. For instance, the condition of the meat (sliced or whole) was not consistently specified in the studies, limiting our ability to perform subgroup analysis (Figure 3). The considerable variation in the sample sizes and event rates also posed challenges in conducting subgroup analysis and identifying the sources of high heterogeneity (Figures S2 and S3). Nevertheless, the results provide valuable insights into the prevalence of *Campylobacter* in poultry and meat, aiding in understanding the trends and high-risk foods.

A study conducted in Brazil also showed that *C. jejuni* was more prevalent in poultry (28.8%) compared with *C. coli* (15.6%) [69]. In a Netherlands case study, consuming poultry and undercooked meat was associated with more *C. jejuni* infections than *C. coli* infections [69]. Usually, there are more cases found related to *C. jejuni*, but cases also exist where *C. coli* surpasses *C. jejuni*, as a study in Argentina showed that *C. coli* (59%) was more prevalent than *C. jejuni* (41%) in slaughterhouse samples [70]. The variation in prevalence between two species could be due to factors such as seasons, geography, and the evolutionary forces of recombination [71,72].

Studying food, its environment, and processing units is crucial for comprehensively understanding pathogen contamination risks. It allows for identifying contamination sources, assessing transmission pathways, evaluating overall risk, and developing effective intervention strategies [73]. A notable example is the 2017 outbreak of *C. jejuni* in Seoul, Korea, where environmental factors and improper handling were implicated as potential causes [8]. Chai et al., (2008) showed that up to 38.2% of *C. jejuni* was transferred from vegetables to wash water, up to 47.2% from wash water to cucumbers, and up to 73.3% from cutting boards to cucumbers, highlighting the importance of environmental factors [74]. In Figure 4, the forest plot shows the high prevalence of *C. jejuni* through handling and equipment sources and contamination through feces. The data in this study (Figure 4) suggest that, given the high prevalence of *Campylobacter* in environmental sources, there could be high contamination in final food products, which, upon consumption, may pose a threat to public health. Although these results show the high contamination risks, a lack of enough studies puts a limitation on finding the ultimate source.

*Campylobacter* contamination sources have been the subject of extensive research because of the prevalence of *Campylobacter* infections worldwide. Poultry, especially chicken and turkey, is a well-documented reservoir of *Campylobacter* species, with high prevalence rates reported in many countries [75]. *Campylobacter* colonization in poultry can be attributed to the gut microflora of these birds, which serves as a natural reservoir. Additionally, improper handling, cross-contamination during processing, and the consumption of undercooked poultry products have all been implicated in *Campylobacter* infections [10].

Moreover, *Campylobacter* can also contaminate water sources, posing a risk to individuals who consume untreated or contaminated water [76]. The primary sources of *Campylobacter* contamination in surface water have been identified as wild birds and poultry, although their influence varies based on factors such as the type of water body, the time of year, and the concentrations of local poultry and ruminant populations [77]. Research has revealed that isolates from poultry exhibit a prolonged survival period compared with other sources, suggesting a critical role in the transmission of *Campylobacter* through water sources [78]. Even in our meta-analysis, river and lake water, chicken wash water, and others revealed a significant amount of positive *Campylobacter* cases, with *C. jejuni* being the predominant species. Notably, a study on waterborne-outbreak-associated *C. jejuni* provided insight into how bacteria originating from cattle manure can infiltrate groundwater, leading to the contamination of water supplies [79]. Understanding these diverse contamination sources is crucial for the prevention and control of *Campylobacter* infections, and ongoing research seeks to elucidate the complex dynamics involved in *Campylobacter* transmission.

## 5. Conclusions

This review comprehensively examined the prevalence of *Campylobacter in* South Korea in poultry, meat, and environmental contexts. The results highlighted ducks as a high-risk food source, corroborating previous research showing higher antibiotic resistance than chickens. The widespread presence of *Campylobacter* species across various meat types and processing settings indicates the urgent need for stringent hygiene measures throughout the production chain. The diverse findings emphasize the significance of tailored control strategies in mitigating the risk of *Campylobacter* contamination in meat products, thereby safeguarding public health and emphasizing the importance of continuous monitoring and intervention efforts in the meat industry. The insights derived from this analysis can serve as a foundation for shaping future strategies in food safety management. By understanding the prevalence and distribution of *Campylobacter* in meat and processing environments, regulatory bodies and industry stakeholders can design interventions to target specific sources of contamination. This knowledge can guide the development of more effective hygiene protocols, surveillance programs, and risk assessment models, reducing the incidence of foodborne illnesses associated with *Campylobacter.*

## Figures and Tables

**Table 1:** Characteristics of studies with the prevalence of *Campylobacter* spp. in South Korea.

Reference	Sampling Period (YYYY. MM)	Sample Group	Sample	Total Sample Size	*Campylobacter* spp. (No. of Positive Samples)	*C. jejuni * (No. of Positive Samples)	*C. coli* (No. of Positive Samples)	Detection Methods	
								Enrichment Medium	Selective Medium
Kang et al., 1999 [31]	1996.03–1998.10	Food	Beef	300	45	0	0	VTP-Brucella FBP broth	Campy BAP
			Beef (frozen)	50	1	0	0		
			Pork	288	68	0	0		
			Pork (frozen)	50	1	0	0		
			Chicken	300	70	0	0		
			Chicken (frozen)	50	0	0	0		
Kang et al., 1985 [32]	1985.03–1985.05	Food	Chicken	449	79	79	0	VTP-Brucella FBP broth	Campy BAP
		Environment	Feces of chicken	278	67	67	0		
Kim et al., 2013 [33]	2010.09–2010.12	Environment	Feces of duck	430	112	112	0	CEB	CBFA
Kim and Chong, 1996 [34]	1996.01–1996.08	Food	Chicken	313	190	0	0	-	BM
Kim et al., 2020 [35]	2015	Food	Chicken	30	23	12	11	2 × BD	PA
			Chicken (cut)	30	23	18	4		
Kim et al., 1986 [36]	-	Environment	River and lake waters	48	1	1	0	BM	BM
Na et al., 2007 [37]	-	Environment	Feces of chicken	80	0	0	0	HB	Modified CBFA
			Chicken wash water	20	9	0	0		
Park et al., 2002 [38]	2000.05–2000.10	Food	Beef	145	0	0	0	SCB	CBFA
Lee et al., 2015 [39]	2013.02–2014.10	Food	Chicken	204	30	15	15	BD	*Campylobacter* agar base, blood agar
Yang et al., 2014 [40]	2009.06–2010.01	Environment	Feces of duck	117	99	93	6	BB	MCCDA-PA, blood agar
Oh et al., 1988 [41]	1987.06–1987.09	Environment	Feces of chicken	120	41	41	0	BB	Campy BAP
		Food	Chicken	20	9	9	0		
		Food	Chicken (frozen)	20	11	11	0		
		Environment	Chilling water	20	12	12	0		
		Environment	Knife	20	9	9	0		
Woo, 2005 [42]	1996.03–1996.10	Food	Chicken	25	16	0	0	-	-
Woo, 2007 [43]	2007	Food	Chicken	115	43	43	0	-	-
Hong et al., 2002 [44]	1997	Food	Pork	296	24	0	0	-	Campy brucella agar
An et al., 2018 [45]	2012.08–2013.09	Environment	Feces of cattle	266	68	68	0	-	MCCDA
			Bedding sample of cattle	32	3	3	0		
Cho et al., 2012 [46]	2011.02–2011.10	Food	Beef	52	0	0	0	BD	CBFA
			Pork	62	0	0	0		
			Chicken	41	0	0	0		
Chon et al., 2018 [47]	2014.06–08, 2014.12–2015.02	Food	Chicken	120	38	0	0	2 × blood-free BD	MCCDA
			Duck	120	93	0	0		
Chon et al., 2016 [48]	2015.01–2015.02	Food	Pork by-product	95	5	0	0	BD	MCCDA
			Chicken by-product	159	8	0	0		
Han et al., 2007 [49]	2004.02–2004.09	Food	Chicken	265	181	100	94	BD	Abeyta–Hunt–Bark agar
Hong et al., 2007 [50]	2001.09–2006.04	Food	Chicken	270	220	140	170	BD	CBFA
			Pork	250	3	3	3		
			Beef	250	4	0	4		
Hong and Lim, 2015 [51]	-	Environment	Dishcloth	50	0	0	0	Modified BD	MCCDA
			Chopping board	50	0	0	0		
			Drawer of Refrigerator	50	0	0	0		
Jeong et al., 2017 [52]	-	Food	Beef	80	1	1	0	-	MCCDA + Preston enrichment broth
Kim et al., 2019 [53]	2016.12–2017.03 2017.04–06	Food	Chicken	133	67	51	29	BD	PA
			Duck	61	38	30	19		
Kim et al., 2010 [54]	2004–2008	Food	Poultry meat (domestic)	475	375	219	156	PB	CBFA
			Poultry meat (imported)	867	217	173	44		
Kim et al., 2017 [55]	2013.12–2014.03	Food	Chicken	124	37	0	0	2 × BD	PA
Lee et al., 2015 [56]	-	Food	Pressed ham with antimicrobials	80	0	0	0	BD	Modified CCDA-PA and MCCDA
			Pressed hams without antimicrobials	80	0	0	0		
			Fermented–cured hams	40	0	0	0		
Lee et al., 2017 [57]	2014.06–08, 2014.12–2015.02	Food	Chicken	152	15	0	0	2 × blood-free BD	MCCDA
			Duck	154	30	0	0		
Lee et al., 2019 [23]	-	Food	Patties	96	0	0	0	-	Modified CCDA-PA
			Meatballs	73	0	0	0		
			Cutlets	55	0	0	0		
Choi et al., 2012 [58]	2010.01	Environment	Feces of pig	100	55	33	22	-	PA
Wei et al., 2016 [59]	2013.01–03	Food	Chicken	80	47	42	5	2 × BD	MCCDA
			Duck	52	0	39	13		
			Duck (sliced)	54	50	43	6		
Park et al., 2010 [60]	2005–2009	Food	Beef (domestic)	630	1	1	0	PB	CBFA
			Pork (domestic)	644	1	1	0		
			Chicken (domestic)	609	187	125	62		
			Duck (domestic)	70	32	18	14		
			Beef (imported)	711	0	0	0		
			Pork (imported)	943	1	1	0		
			Chicken (imported)	546	109	83	26		

Abbreviations: YYYY= year, MM= month, BD = Bolton broth, MCCDA = modified charcoal cefoperazone deoxycholate agar, CEB = *Campylobacter* enrichment broth, BB = Brucella broth, PB/PA = Preston broth/Preston Agar, BM = Butzler medium, SCB = Skirrow’s *Campylobacter* selective broth, HB = Hunt broth, CBFA = *Campylobacter* blood-free agar, Campy BAP = BD *Campylobacter* agar + ASB, VTP = vancomycin–trimethoprim–polymyxin B, FBP = Brucella–fructose-1,6-bisphosphate.

**Table 2:** Meta-analysis results for the overall study and each food type and environment.

Sample Type		No. of Studies	Pooled Prevalence and 95% Interval			I^2^ (%)	*p*-Value
			Pooled Prevalence (%)	Lower Limit (%)	Upper Limit (%)		
Overall		31	23.38	16.78	31.58	98%	<0.001
Food	Chicken	22	36.17	26.44	47.19	97%	<0.001
	Duck	6	70.46	42.80	88.38	96%	<0.001
	Beef	8	0.99	0.20	4.71	90%	<0.001
	Pork	8	2.10	0.67	6.35	94%	<0.001
Environment	Feces	7	36.33	22.62	52.68	96%	<0.001
	Washing water	3	27.69	6.05	69.47	86%	0.001
	Equipment	5	4.99	0.76	26.41	84%	<0.001

**Table 3:** Prevalence of *C. jejuni* and *C. coli* in duck.

Author	Total Sample Size	Total Positive Samples (%)	*C. jejuni* (%)	*C. coli* (%)
Wei et al., 2016 [59]	52	52 (100)	39 (75.0)	13 (25.0)
Wei et al., 2016 [59]	54	50 (92.6)	43 (79.6)	6 (11.1)
Park et al., 2010 [60]	70	32 (45.7)	18 (25.7)	14 (20.0)

**Table 4:** Prevalence of *C. jejuni* and *C. coli* in chicken.

Author	Total Sample Size	Total Positive Sample (%)	*C. jejuni* (%)	*C. coli* (%)
Kim et al., 2019 [53]	67	67 (100)	51 (76.1)	29 (43.3)
Wei et al., 2016 [59]	80	47 (58.8)	42 (52.5)	5 (6.3)
Park et al., 2010 [60]	609	187 (30.7)	125(20.5)	62 (10.2)
Park et al., 2010 [60]	546	109 (20.0)	83 (15.2)	26 (4.8)
Kang et al., 1985 [32]	449	79 (17.6)	79 (17.6)	0 (0.0)
Kim et al., 2020 [35]	30	23 (76.7)	12 (40.0)	11 (36.7)
Kim et al., 2020 [35]	30	22 (73.3)	18 (60.0)	4 (13.3)
Lee et al., 2015 [56]	204	30 (14.7)	15 (7.4)	15 (7.4)
Oh et al., 1988 [41]	20	9 (45.0)	9 (45.0)	0 (0.0)
Oh et al., 1988 [41]	20	11 (55.0)	11 (55.0)	0 (0.0)
Woo, 2007 [43]	115	43 (37.4)	43 (37.4)	0 (0.0)

## Data Availability

Data are contained within the article.

## Supplementary Materials

The following supporting information can be downloaded at https://www.mdpi.com/article/10.3390/microorganisms11112722/s1: Figure S1: Risk of bias assessment for all included studies presented as the percentage of bias risk for each question. Figure S2: Funnel plot of the overall study for the prevalence of *Campylobacter* in South Korea. Figure S3: Funnel plot of each food type for the prevalence of *Campylobacter* in South Korea: (a) chicken, (b) duck, (c) pork, (d) beef.
