# Supplementary material for: Systematic Review and Meta-Analysis of Campylobacter Species Contamination in Poultry, Meat, and Processing Environments in South Korea
Source: Microorganisms. 2023 Nov 7;11(11):2722. doi: 10.3390/microorganisms11112722 (PMC10673067; doi:10.3390/microorganisms11112722)
Supplement: Supplementary file 1 [file microorganisms-11-02722-s001.zip › microorganisms-2696015-SI.pdf]

Supplementary Figures

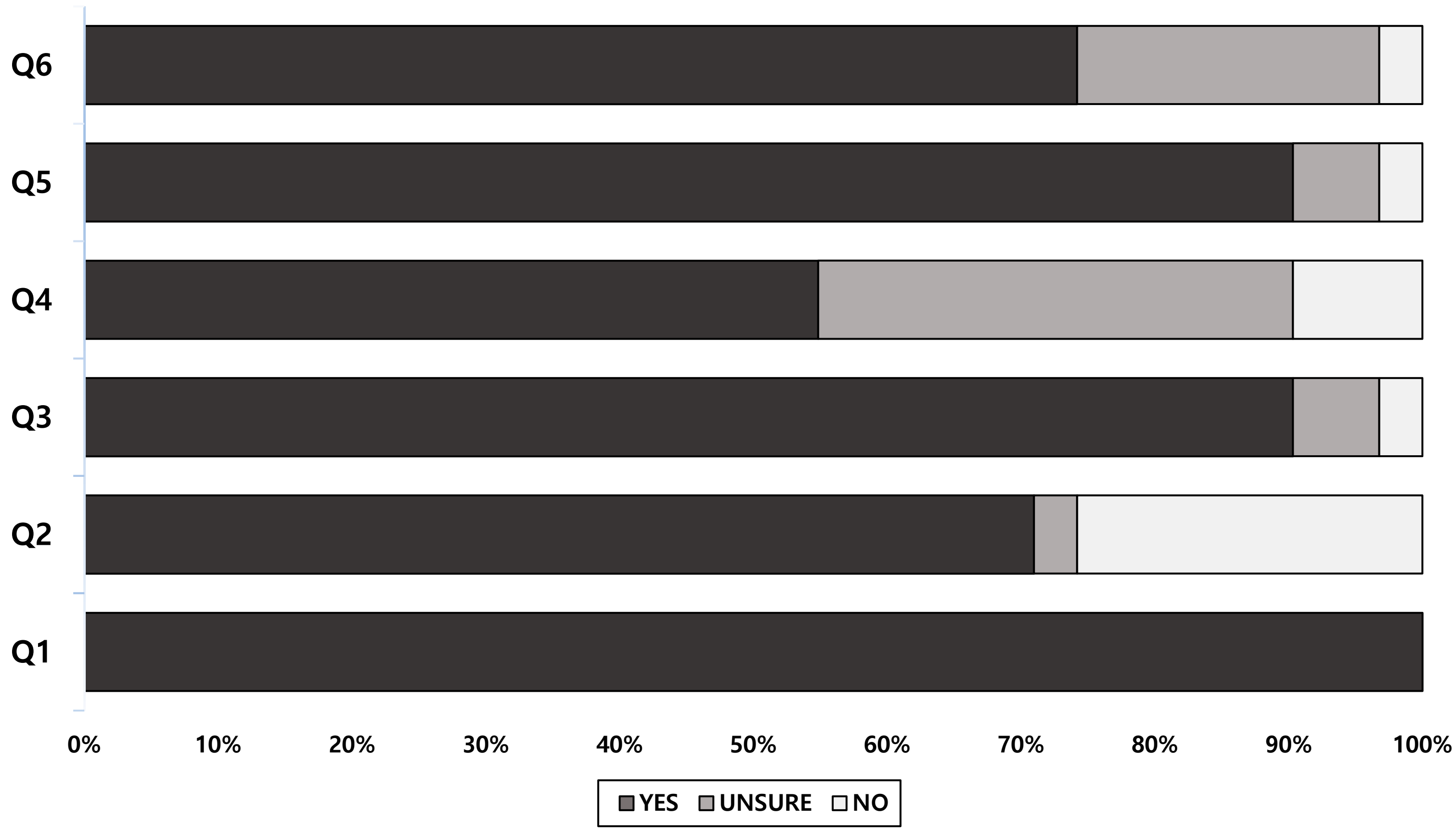

Figure S1. Risk of bias assessment for all included studies presented as the percentage of bias risk for each question.

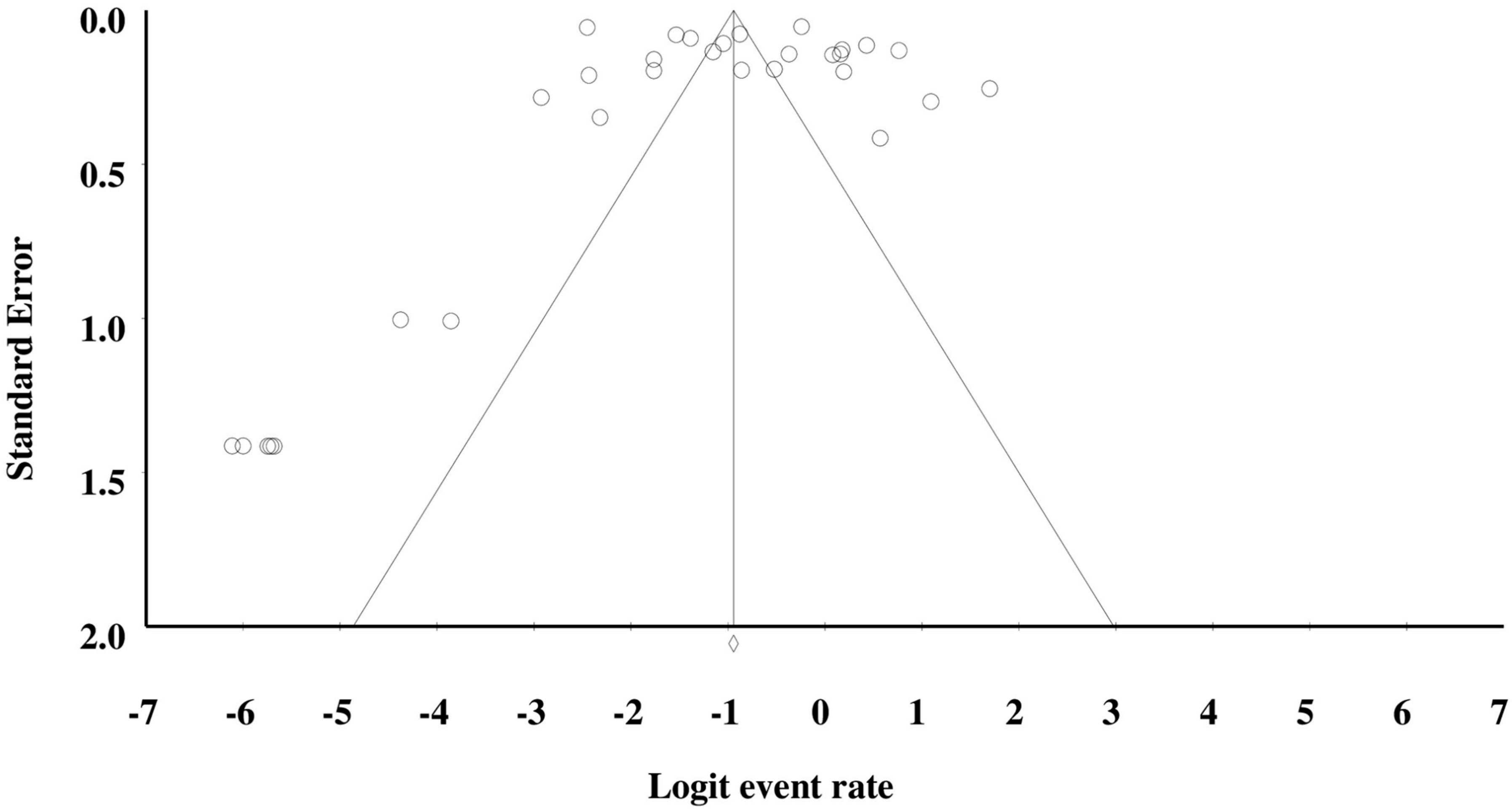

Figure S2: Funnel plot on the overall study for the prevalence of *Campylobacter* in South Korea.

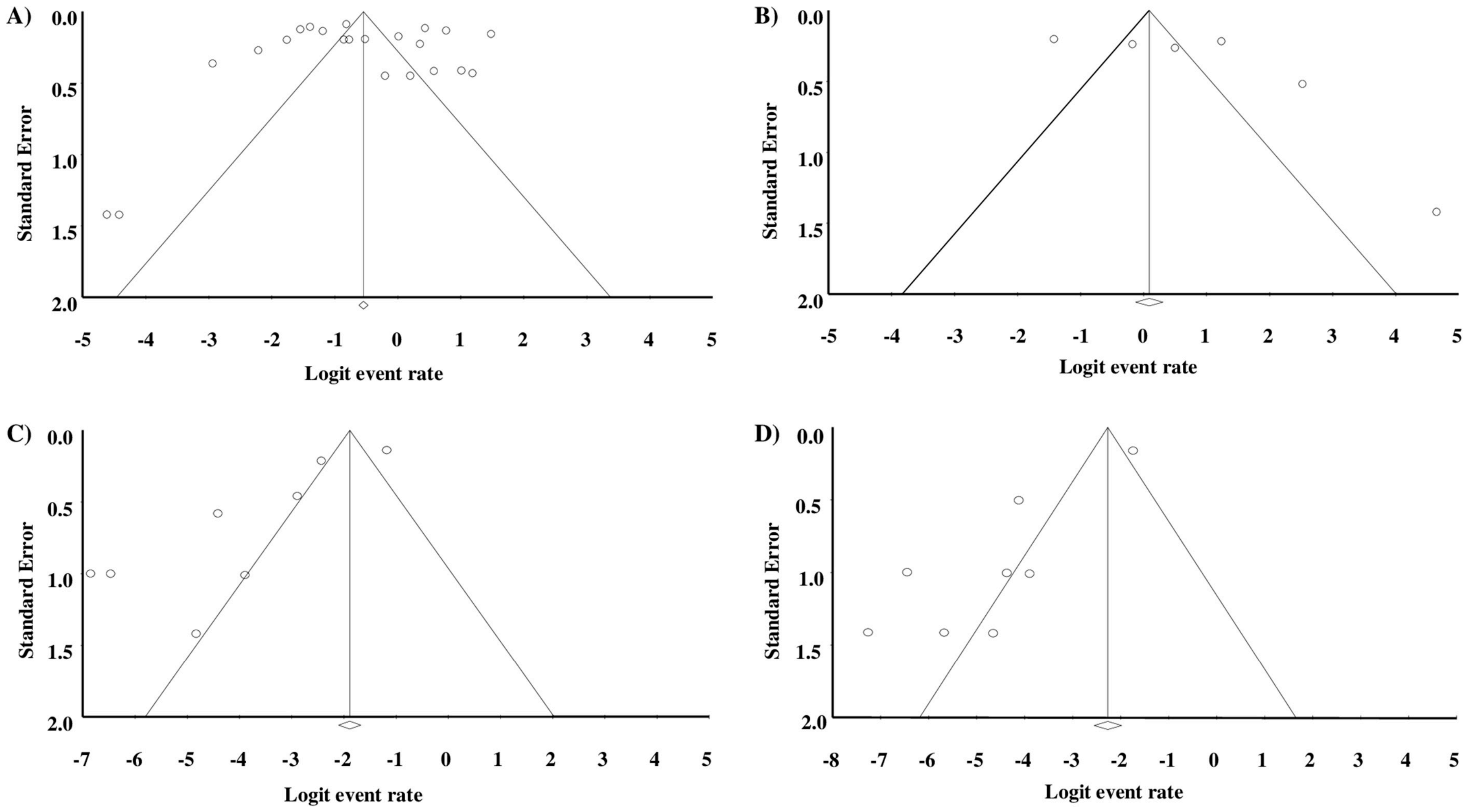

Figure S3: Funnel plot on each food type for the prevalence of *Campylobacter* in South Korea: (**a**) chicken, (**b**) duck, (**c**) pork, (**d**) beef.
